# Mobile Virtual Reality Versus Mobile 360° Video to Promote Enrollment in the Diabetes Prevention Program Among Hispanic Adults: Pilot Study

**DOI:** 10.2196/26013

**Published:** 2022-03-17

**Authors:** Bryan Gibson, Sara Simonsen, Jakob D Jensen, Leah Yingling, Julia Schaefer, Vishnu Sundaresh, Yue Zhang, Roger Altizer

**Affiliations:** 1 Department of Biomedical Informatics School of Medicine University of Utah Salt Lake City, UT United States; 2 College of Nursing University of Utah Salt Lake City, UT United States; 3 Department of Communication University of Utah Salt Lake City, UT United States; 4 Department of Endocrinology School of Medicine University of Utah Salt Lake City, UT United States; 5 Division of Epidemiology Department of Internal Medicine University of Utah Salt Lake City, UT United States; 6 Therapeutic Games and Applications Lab University of Utah Salt Lake City, UT United States

**Keywords:** diabetes prevention, virtual reality, risk, perception, diabetes, type 2 diabetes, mobile phone, prediabetes, prevention, VR, enrollment, pilot study, video

## Abstract

**Background:**

Hispanic adults are at increased risk of developing type 2 diabetes. The Diabetes Prevention Program (DPP) reduces the risk of developing type 2 diabetes; however, the rate of enrollment is very low.

**Objective:**

The goal of this pilot project was to determine whether presenting brief motivational mobile videos in virtual reality vs 360° video has differential effects on risk perceptions and enrollment in the DPP.

**Methods:**

Adults with prediabetes were recruited at a clinic serving a low-income Hispanic community. After consenting, the participants completed a baseline survey that collected information about demographics and risk perceptions. All participants then viewed 2 videos. Per random assignment, the videos were presented either using the participant’s smartphone alone (360° video) or were viewed with their smartphone in a virtual reality (VR) cardboard headset. Two weeks later, a follow-up survey collected measures of enrollment in the DPP, risk perceptions, health literacy, the importance of contextual factors related to the decision of whether to enroll in the DPP (eg, distance to the class), and qualitative feedback on the interventions. We used logistic regression to determine whether enrollment in the DPP differed by intervention mode, while accounting for health literacy and contextual factors related to the DPP. We used unpaired *t* tests to examine differences in change in risk perceptions between groups. Paired *t* tests were used to examine within-subject changes in risk perceptions.

**Results:**

A total of 116 participants provided complete data. Most participants were middle-aged (mean age 44.6 years; SD 11.9) Hispanic (114/116), female (79/116), with low health literacy (mean score 12.3/20; SD 3.4). Enrollment in the DPP was 44/116 (37.9%) overall but did not differ by group (odds ratio for enrolling in VR group 1.78, 95% CI 0.75-4.3; *P*=.19). Individuals who rated the distance needed to travel to attend the DPP as more important were less likely to enroll in the DPP (odds ratio 0.56, 95% CI 0.33-0.92; *P*=.03). Risk perceptions did not differ by group (mean change in 360° video group -0.07, mean change in VR group 0.03, *t*=0.6, *P*=.54) and did not change within subjects (mean 0.02, *t*=0.21, *P*=.83). Participant feedback suggested that the videos are emotionally engaging and educational.

**Conclusions:**

The videos presented in 360° video and mobile VR had equal efficacy in promoting enrollment in the DPP. Future work to rigorously evaluate this intervention, its mechanism of action, and potential moderators of the efficacy are discussed.

## Introduction

More than 42% of Hispanic adults in the United States have prediabetes, placing them at increased risk of type 2 diabetes mellitus (T2DM) [1]. Extensive evidence has demonstrated that moderate lifestyle changes can reduce the progression from prediabetes to T2DM by 58% [2]. To address this epidemic, the Centers for Disease Control and Prevention have established the national Diabetes Prevention Program (DPP) [3]. The DPP is a yearlong lifestyle change program and has been shown to be effective in reducing the risk of developing T2DM [4]. However, through 2019, only 0.4% of the 88 million adults in the United States with prediabetes have enrolled in the DPP [4], and only 8.6% of these enrollees are Hispanic [5]. Clearly, there is need for scalable interventions that increase enrollment in the DPP among Hispanic adults.

Currently, most individuals with prediabetes who enroll in the DPP are identified by their primary care provider (PCP), counseled regarding the benefits of the program, and then referred. Studies of provider referrals to the DPP have reported variable DPP enrollment rates, ranging from 8% to 19% [6-8]. While provider counseling and referral is a useful means to promote DPP enrollment, there are 2 significant limitations to this approach. First, most providers do not currently counsel their patients about lifestyle changes and weight loss [9,10]. Adding diabetes prevention counseling to PCPs’ already heavy workload may only exacerbate their perceptions of lack of time. Additionally, many PCPs lack training in counseling, leading to missed opportunities [11,12]. Second, DPP enrollment program that is based on a clinical encounter will necessarily miss the many individuals who do not seek primary care in a given year [13], a problem that may be more prevalent among Hispanic adults who access primary care less frequently than adults of other ethnicities [14]. Thus, we sought to compare 2 approaches to promoting DPP enrollment that do not require provider counseling or referral.

In this pilot project, we compared the effects of mobile 360° video vs mobile virtual reality (VR) on participants’ risk perceptions and enrollment in the DPP. The participants were randomized to 1 of the 2 following study groups: mobile 360° video and mobile VR. The participants in both groups viewed 2 videos that contained the same content in different delivery modalities. The videos demonstrate the possible negative future complications of diabetes. Those assigned to mobile 360° video watched the videos on their smartphone (the viewer moved their phone to “look around” the world of the movie) while those assigned to mobile VR watched the videos using their smartphone inside a cardboard VR headset with headphones. The goal of this project was to determine if there was a difference in DPP enrollment rates between those who watched the videos using mobile 360° technology vs mobile VR. While we expected that VR would lead to greater changes in risk perceptions and higher enrollment in the DPP than the same videos viewed as 360° video, the study was not designed to measure the possible mediators of that effect. Future studies are planned to examine this question.

The rationale for comparing mobile 360° video vs mobile VR is twofold. First, the question is largely unaddressed; prior research comparing 360° video and immersive VR is very limited [15,16] and has not addressed health risk presentation or risk perceptions in individuals at risk of chronic disease. Second and more importantly, information on any differential effects of these 2 modes of intervention delivery would be valuable in the design of future interventions to promote health behaviors, particularly those that seek to target low-income, at-risk communities. Mobile 360° videos are highly scalable (ie, could be texted to anyone with a smartphone) while VR requires a headset and headphones that many low-income individuals may not have access to or may not be comfortable using without assistance.

## Methods

### Conceptual Framework

The reasons an individual may enroll (or not enroll) in the DPP are multifactorial. First, only 15% of Americans with prediabetes are aware they have this health condition [17]. Secondly, many individuals with prediabetes lack knowledge about appropriate health behavior changes (eg, increasing physical activity and weight loss) needed to prevent T2DM [17,18]. In addition, many individuals with prediabetes have an inaccurate understanding of the risks associated with developing T2DM and its complications [19]. Finally, even individuals who are aware that they have prediabetes and understand the risks may not enroll in the DPP because of practical barriers such as the cost of enrollment, limited time for participation (22 sessions over 12 months), and difficulty with travel to and from DPP sessions [20].

**Figure 1:**
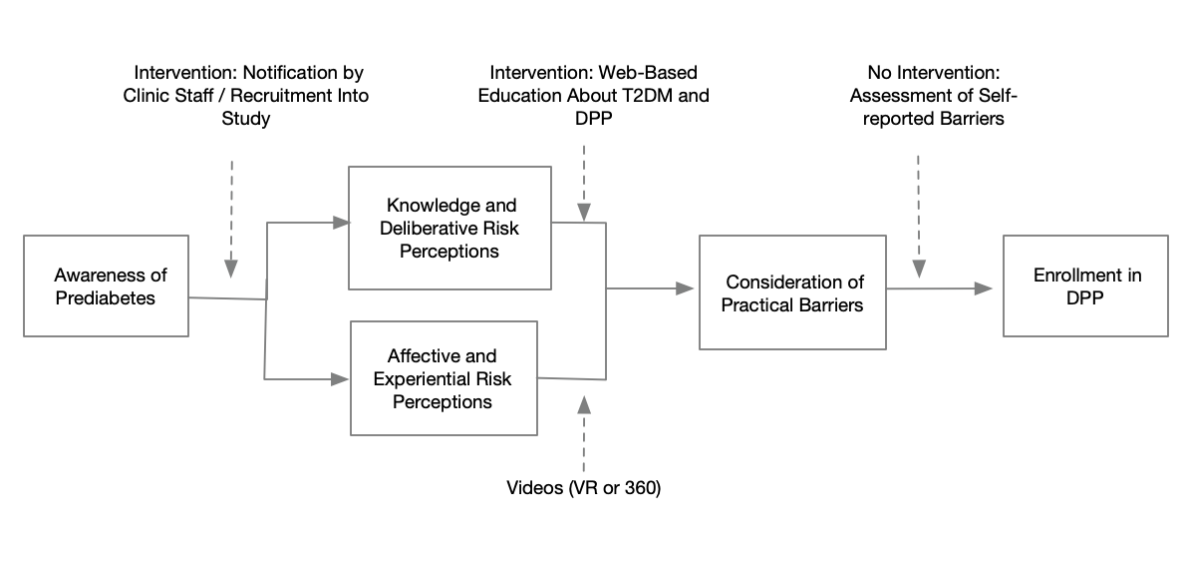
Conceptual framework. DPP: Diabetes Prevention Program; T2DM: type 2 diabetes mellitus; VR: virtual reality.

Our intervention addressed or measured (for use as a covariate) each of the factors shown in Figure 1. First, to address low awareness of prediabetes, all participants were called by the clinic’s health coach, were informed that they have prediabetes, and were asked if they would like to participate in the study.

Second, to address low risk perceptions, individuals were randomized to either the VR or 360° version of our videos. The proposed mechanism of action for these videos was based on the tripartite model of risk perception [21], which divides risk perceptions into deliberative risk perceptions (ie, the individual’s estimates of the likelihood of developing a condition), affective risk perceptions (ie, the individual’s level of worry about a particular risk), and experiential risk perceptions (ie, how easy it is to imagine developing a condition). The videos were hypothesized to increase the participants’ affective and experiential risk perceptions, and this would motivate them to enroll in the DPP. Third, to address limited knowledge about prediabetes, T2DM, and the DPP, all participants were sent a link to a website [22], which includes the 3 following components: a self-assessment of risk (using the American Diabetes Association risk score, hypothesized to change deliberative risk perceptions), didactic pages about prediabetes and T2DM, and didactic pages about the DPP and its benefits.

Finally, while this pilot study did not have the resources to intervene on the practical barriers to enrolling the DPP, we measured individuals’ reports of these barriers (eg, cost and time for participation) for use as covariates when estimating the videos’ efficacy.

### Description of Videos

In this pilot project, all participants watched the same 2 immersive videos. These were developed by our research team after an extensive process of co-development with community members at risk of diabetes. The first video demonstrates how one's vision worsens over years with diabetic eye disease. The second video is a first-person narrative of an individual who progressively develops T2DM, oral health issues, and heart disease. Both videos conclude with a positive message that enrolling in the DPP may aid in preventing these potential negative outcomes. Both videos include a male or female voice-over in either Spanish or English (selected by the participant at the start of the video).

### Ethical Considerations

The study protocol was approved by the University of Utah Institutional Review Board prior to study initiation (approval number: 00115941). Informed consent (in English or Spanish) was obtained from all participants, via consent cover letter, prior to data collection.

### Recruitment and Settings

This project was conducted in partnership with the Midvale Community Building Community Clinic. This clinic serves a primarily low-income Hispanic population in Midvale, Utah. This clinic offers the DPP to its patients at no cost.

### Procedures

Potential participants were identified in the clinic’s electronic health record by the clinic’s health coach (using hemoglobin A_1c_ values 5.7-6.4). They were then informed via telephone that they have prediabetes and asked if they were willing to participate in the study. During this call, individuals were screened to ensure they owned a smartphone, which was required for study participation.

Individuals who agreed to participate were contacted by the research assistant to meet in person at the clinic. During the in-person meeting, the study was explained, and the participant underwent informed consent. All study materials and the videos were provided in either English or Spanish according to the participant’s preference.

The baseline questionnaire collected information on demographics and a yes or no question on whether the individual had prediabetes (a check on whether they understood the notification from the clinic’s health coach); it also included an 18-item validated measure of risk perception [23]. After completion of the baseline questionnaire, the participants were randomized to receive either the mobile 360° video or the mobile VR experience. Individuals in the VR group were provided with a cardboard headset and headphones and watched the videos privately in the clinic conference room. Individuals in the mobile 360° video group watched the videos on their smartphone, privately in the clinic conference room. Technical issues that the participants experienced with either platform were noted for future refinement of the intervention. Prior to leaving the clinic, the participants were given a flier for the DPP offered by the clinic, which included enrollment instructions. Within 2 days of the baseline meeting, each participant was sent an SMS message with a link to the “doihaveprediabetes” website [22], an educational website that provides information on prediabetes, T2DM, and the DPP.

Two weeks later, the participants were sent a follow-up survey to their mobile phone, which included questions about whether they enrolled in the DPP, a repeat assessment of their risk perceptions, qualitative feedback about the videos (VR or 360) and informational website, as well as a standardized measure of health literacy [24]. The questionnaire concluded with a set of 8 Likert-type questions about the importance of practical barriers or facilitators to enrollment in the DPP, including the following: language the DPP is offered in; availability of childcare at the DPP; accessibility of the DPP in terms of location; time requirements and scheduling [25]; and the desire to participate in the DPP if it were delivered by internet. To compensate for participation in this trial, the participants were emailed a US $75 electronic gift card.

### Analysis

To test whether there were significant differences in the distributions of the participants’ demographics for completers (participants who at least provided data on DPP enrollment in the follow-up survey) vs noncompleters (participants who did not start the follow-up survey) and between completers randomized to VR vs 360° video, we used chi-squared tests for categorical variables and *t* test for continuous variables.

The primary outcome of interest was self-reported enrollment in the DPP. We used logistic regression to compare the effects of the 2 modalities of video delivery on the likelihood of enrollment in the DPP. This model adjusted for any baseline difference in demographics between groups, a dichotomous variable that indicated whether the individual was aware that they had prediabetes, and the participants’ rating of the importance of practical factors that affected their decision of whether to enroll as covariates.

To test whether the videos caused changes in risk perception, we first used paired *t* tests to determine whether there were significant within-subject changes in risk perceptions. We then used an unpaired *t* test to compare changes in risk perception by intervention modality. An exploratory mediation analysis was planned if there had been significant changes in risk perceptions. All analyses were conducted using statistical programming language R (R Foundation for Statistical Computing) [26].

## Results

### Participation

Approximately 240 patients were contacted by the clinic’s health coach and notified that they had prediabetes. A total of 209 consented and participated in baseline assessments, and 116 participants provided complete follow-up data. The majority of the loss to follow-up occurred in the first few months of data collection because we originally compensated participants with a gift card at the end of the baseline assessment rather than after the follow-up assessment. Some data were also lost because some questions were not mandatory in the online follow-up questionnaire (both issues were addressed about half of the way through the pilot).

Table 1 provides the measured demographics of individuals who were randomized to VR vs 360° video; there were no significant differences in demographics between the groups.

**Table 1:** Demographics of participants by intervention group among those who completed baseline and follow-up interviews.

Characteristics			Values						
			VR^a^ group		360° group		Statistic		*P* value
**Sex (baseline survey), n (%)**							1.9^b^		.38
	Female	36 (64)		43 (72)					
	Male	20 (36)		16 (27)					
	Prefer not to answer	0 (0)		1 (1.7)					
**Language (baseline survey), n (%)**							0.8^b^		.36
	English	12 (21.4)		8 (13.3)					
	Spanish	44 (78.6)		52 (87.7)					
**Race (baseline survey), n (%)**							7.7^b^		.10
	White	44 (78.6)		33 (55)					
	African American	0 (0)		2 (3.3)					
	Native American	0 (0)		0 (0)					
	Asian	0 (0)		1 (1.7)					
	Pacific Islander	0 (0)		1 (1.7)					
	Other	12 (21.4)		24 (40)					
**Ethnicity (baseline survey), n (%)**							2.0^b^		.36
	Hispanic/Latino	55 (98.2)		59 (98.3)					
	Not Hispanic/Latino	1 (1.8)		1 (1.7)					
**Aware of prediabetes (follow-up survey), n (%)**							0.03^b^		.98
	Yes	48 (85.7)		53 (88.3)					
	No	6 (10.7)		6 (10)					
	I don’t know	2 (3.6)		1 (1.7)					
Health literacy score (follow-up survey), mean (SD)			12.7/20 (3.4)		11.9/20 (3.4)		-1.2^c^		.22
Age (baseline survey) (years), mean (SD)			43.9 (13.6)		45.2 (10.3)		-0.57^c^		.56

^a^VR: virtual reality.

^b^Chi-square test.

^c^*t* test.

### DPP Enrollment

A total of 116 participants provided data on DPP enrollment; overall enrollment in the DPP was 44/116 (37.9%). Enrollment among those randomized to VR was 25/56 (44.6%), while enrollment among those randomized to the 360° video was 19/60 (31.6%). To determine if the presentation modality was associated with differential enrollment rates after adjusting for relevant covariates, we created a logistic regression model with enrollment as the outcome and intervention modality, awareness of prediabetes (a check on whether they understood the notification from the clinics health coach), and participants’ ratings of the importance of factors that might affect their decision of whether or not to enroll (“which factors were important in your decision of whether or not to enroll in the DPP?” distance, time cost, etc) as covariates. Table 2 shows the results of that model.

**Table 2:** Model results from logistic regression predicting Diabetes Prevention Program enrollment.

Criteria	Odds ratio	Lower 95% CI	Upper 95% CI	*P* value
VR^a^	1.78	0.75	4.30	.19
Health literacy score	1.08	0.95	1.24	.22
Aware of prediabetes	2.06	0.54	9.20	.30
Importance of language DPP^b^ is offered in	0.78	0.49	1.23	.30
Importance of distance to DPP	0.56	0.33	0.92	.03
Importance of time required for DPP	1.55	0.94	2.66	.09
Importance of availability of DPP via internet	1.00	0.56	1.71	.98
Importance of cost of DPP	1.05	0.64	1.73	.83
Importance of availability of childcare through DPP	1.14	0.73	1.80	.56
Importance of motivation to change lifestyle	0.73	0.42	1.24	.25

^a^VR: virtual reality.

^b^DPP: Diabetes Prevention Program.

### Risk Perceptions

Complete pre-post risk perception data were available for 96 people. Table 3 shows the prevideo and postvideo scores for total risk perception score, and for each component of the risk perception scale. Neither the total risk perception score nor its components changed significantly. *T* tests comparing the changes by intervention modality found no significant difference in risk perception changes between groups: total risk score (*t*=-0.6; *P*=.54), deliberative risk score (*t*=-0.6; *P*=.54); affective risk score (*t*=0.44; *P*=.65); and experiential risk score (*t*=-1.6; *P*=.10)

**Table 3:** Risk perception pre video and post video by intervention group.

Risk perception score component	VR^a^ group (n=49)		360° video group (n=47)	
	Prevideo, mean (SD)	Postvideo, mean (SD)	Prevideo, mean (SD)	Postvideo, mean (SD)
Total score	3.43 (0.74)	3.46 (0.76)	3.61 (0.75)	3.54 (0.70)
Deliberative component score	2.98 (0.87)	2.98 (0.86)	2.85 (0.96)	2.75 (0.84)
Affective component score	3.63 (1.63)	3.60 (1.72)	4.12 (1.72)	4.12 (1.80)
Experiential component score	3.69 (0.69)	3.82 (0.75)	3.86 (0.72)	3.74 (.73)

^a^VR: virtual reality.

### Qualitative Feedback

In the follow-up survey, depending upon the video modality they received, the participants were asked “What did you think of the VR/Mobile 360° video?” Many participants provided extremely short feedback such as “good” or “educational.” Textbox 1 provides a sampling of the more detailed comments that were provided, divided into those that were positive and negative in tone.

**Feedback from participants on the videos.**

**Positive response to the videos**

- “Very interesting, it helped me to reflect.”
- “They are very interesting to became conscious on this disease of diabetes.”
- “I was informed with the video, something that I have not seen before. I learned to take care of myself and eat healthy.”
- “It’s a good video on showing the difference on a person having higher risk on diabetes.”
- “Very good information about diabetes prevention.”
- “It is very interesting how I interacted with the program.”
- “I learned to eat healthy to prevent diabetes.”
- “I liked them. They are awesome!”
- “They are very descriptable (sp) about the risk for having high blood sugar.”
- “It really open my eyes.”
- “I thought of how we could lose our sight and even our lives if we do not take care of ourselves adequately, our eating habits and our children's eating habits. And value our health and our family's health. The consequence of our addictive lifestyle of not eating healthy. Our families and ourselves must take care of our health. I would like to learn more.”
- “It was amazing how realistic it made me understand the importance of my health and keeping diabetes at bay.”

**Negative feedback on the videos**

- “They are creative but over dramatics.”
- “Scary.”
- “It was nice but it did hurt my head a bit.”

## Discussion

### Summary of Project and Primary Findings

In this pilot study, we tested whether the presentation of 2 brief motivational mobile phone videos delivered via mobile 360° vs mobile virtual reality had differential effects on risk perceptions and enrollment in the DPP. We found an absolute difference in the enrollment of 13% between groups that, while not statistically significant in this project, might be practically important. We also found that risk perceptions did not differ by modality and did not change for individuals in either group. We believe these results suggest several avenues for further investigation.

While we are unaware of prior research comparing the efficacy of VR to 360° Video on changing individuals’ health beliefs and behaviors, a few studies have tested the use of VR for health behavior change [27]. For example, Ahn et al [28,29] compared the effects of a pamphlet on the health risks of sugary drink consumption alone with a VR simulation of a virtual person gaining weight as a result of regularly consuming soft drinks, with both interventions combined. They found that the combined intervention was more effective than either alone, leading to lower self-reported consumption of soft drinks. Interestingly, they found that the risk perceptions of participants who experienced the VR increased. By contrast, in this study, we found that our immersive videos did not lead to changes in risk perception. Clearly, further research is needed to understand the mechanism of action of immersive videos intended to change beliefs and behaviors.

There are several strengths to this study. First, this study addressed the pragmatic question of whether the greater immersiveness of VR is needed (vs 360° video) for persuasive videos to affect individuals’ health beliefs and behaviors. Second, the study interventions sought to isolate the effect of the mode of video delivery on enrollment by addressing other factors that might affect enrollment. We notified all individuals of their prediabetes to address low awareness of prediabetes; we also sent all participants a website URL to educate them to address their lack of understanding of prediabetes, T2DM, and the DPP. Finally, we measured contextual factors related to the decision about whether to enroll in the DPP (eg, ratings of importance of travel, distance, and cost) for use as covariates in estimating the effect of the intervention.

This study has several limitations. First, our measure of DPP enrollment was based on self-report, leading to the potential for social desirability bias in our results. In addition, we only measured whether people signed up for the DPP, not whether they engaged with the program. In future work, we plan to collect objective data from the DPP program on both enrollment and engagement. Second, this study was an uncontrolled pilot study; therefore, it is possible that simply informing people that they have prediabetes and educating them about the DPP led to their enrollment, independent of the video interventions. However, prior research on notifying individuals that they have prediabetes and educating them about the DPP has found much lower rates of enrollment than we found in this study. For example, as part of a large trial of community-based DPPs, investigators contacted 7500 community members with a letter notifying them that they have prediabetes and educating them about the DPP; they found that that only 1.7% of those who were contacted enrolled in the DPP [7]. The true magnitude of the efficacy of our videos will need to be tested in a controlled trial.

Based on the results of this pilot, we are planning a trial that will compare the efficacy of notification or education alone vs notification or education + VR videos vs notification or education + 360° on objectively measured enrollment and engagement in the DPP among individuals with prediabetes. Future work will also assess multiple potential mechanisms of action for the videos, assessing whether risk perceptions [23], narrative transportation [30], and immersion [31] are associated with the videos’ efficacy and whether the effects of the intervention are moderated by the individuals’ health literacy, numeracy, or practical barriers to enrolling.

### Conclusions

Increasing enrollment in the evidence-based DPP is a national priority. We present a comparison of the presentation of brief motivational mobile phone videos in virtual reality vs 360° video on risk perceptions and enrollment in the DPP. Our results suggest that further work is warranted to determine the replicability of our findings in other populations, to examine the mechanism of action of the videos, and to assess any moderators of their efficacy.

## Abbreviations

DPP: Diabetes Prevention Program
PCP: primary care provider
T2DM: type 2 diabetes mellitus
VR: virtual reality
